# Myocardial Perfusion Imaging and C-Reactive Protein in Myocardial Ischemia: A Retrospective Single-Center Study

**DOI:** 10.3390/life14020261

**Published:** 2024-02-16

**Authors:** Aidonis Rammos, Aris Bechlioulis, Areti Kekiopoulou, Pavlos Kekiopoulos, Christos S. Katsouras, Chrissa Sioka

**Affiliations:** 1 2nd Department of Cardiology, University Hospital of Ioannina, 45110 Ioannina, Greece; 2 Department of Nuclear Medicine, University Hospital of Ioannina, 45110 Ioannina, Greece

**Keywords:** C-reactive protein, ischemia, SPECT, myocardial perfusion imaging, cardiac imaging

## Abstract

Background: Inflammation is an important mechanism in atherosclerosis and plaque formation. C-reactive protein (CRP) is a common inflammatory biomarker associated with the risk of coronary heart disease. We investigated the relationship of CRP with findings from myocardial perfusion imaging (MPI). Methods: In this retrospective study, 102 consecutive patients (mean age 71 years, 68% males) who underwent MPI (for diagnostic reasons or quantification of myocardial ischemia) and CRP determination (upper limit: 6 mg/L) within 1 month from MPI were included. The patients had no infection or recent acute coronary syndrome. Results: The median CRP level was 4 mg/L (2, 10) among the study population. Patients with raised CRP had higher summed stress score (SSS) (*p* = 0.006) and summed rest score (SRS) (*p* = 0.001) and higher risk for SSS > 3 (OR 9.25, 95% CI 2.03–42.13, *p* = 0.001) compared to those with low CRP. The association of SSS and SRS with CRP levels was more evident in patients over 70 years (*p* = 0.027 and *p* = 0.005, respectively). No significant difference in summed difference score was shown. The two groups had no difference in other risk factors (*p* > 0.05 for all comparisons). Conclusion: a high level of CRP was associated with the presence and extent of stress-induced myocardial ischemia in MPI.

## 1. Introduction

Coronary artery disease constitutes the leading etiology of death, suggesting that its effective management is pivotal to reducing morbidity and mortality [1]. In general, atherosclerosis is mainly responsible for cardiovascular disease, with atherosclerotic lesions mainly affecting the large and medium-sized elastic and muscular arteries [2,3]. Inflammation is an important part of the vascular atherosclerotic process, consisting of a slowly progressive inflammatory activity manifested by increasing serum inflammatory cytokines. Inflammatory cytokines such as interleukin 6 (IL-6) stimulate the production of acute-phase reactants, including C-reactive protein (CRP) [4], an easily measured and most widely used biomarker for detecting an acute inflammatory process and monitoring several chronic inflammatory conditions [5,6]. Cumulative evidence indicates that inflammation, both at the focal and systemic levels, plays a role in the disruption of atherosclerotic plaques and acute cardiovascular consequences [7]. Its evaluation in cardiovascular disease appears to be associated with altered CRP and increased cardiovascular risk [8,9,10]. Thus, recent studies tried to elucidate if inflammatory biomarkers such as CRP may identify patients at risk for acute cardiovascular events for early treatment [11].

Apart from its rapid rise and involvement during an acute inflammatory process, an indolent or modest but persistent increase in CRP or high sensitivity CRP (hs-CRP) has been correlated with the presence of coronary artery disease (CAD) and a higher risk of future cardiovascular events in apparently healthy individual [5,12,13,14,15,16]. For example, an increased CRP, even in stable coronary artery disease, was a significant predictor of harmful cardiovascular conditions independently of the specific therapeutic management [17]. In addition, high serum CRP level was associated with the presence but not the degree of CAD in patients with stable angina [18]. Even though an elevated serum CRP level did not correlate with the degree of angiographic abnormalities in coronary arteries, it could possibly help discover new cases of coronary lesions in follow-up examinations [19]. 

Myocardial perfusion imaging (MPI) with single photon emission tomography (SPECT) obtains important information on myocardial perfusion and assesses viable or ischemic myocardial tissue in various conditions, such as angina, non-specific chest pain and macrovascular or microvascular CAD [20,21]. Two decades ago, Lombardi et al., in a small, interesting study, showed that high CRP levels might predict exercise-induced MPI ischemia and patient’s outcome [22]. In another small study, patients with angiographically coronary slow flow (possibly due to endothelial inflammation) had higher hs-CRP levels compared to controls. Moreover, in a proportion of patients with an MPI study, hs-CRP predicted positive MPI results [23]. However, data about the association of CRP with MPI findings are limited [22,23,24,25]. 

In the present study, we aimed to investigate the role of subclinical inflammation in stable coronary artery disease. We investigated the association of serum CRP levels with the presence and extent of ischemia detected after stress-induced MPI SPECT, performed for diagnostic or prognostic reasons. 

## 2. Methods

### 2.1. Study Population

In this retrospective single-center study, a thorough search of all patients who had MPIs performed in the Nuclear Medicine Department of our University Hospital from 1 January 2022 to 31 December 2022 and CRP measurements within one month before or after MPI were included. Patients were subjected to an MPI SPECT study for diagnostic clinical reasons (suspected CAD) or prognostic (quantification of myocardial ischemia) reasons. 

The exclusion criteria included the presence of clinical signs or findings of infection or recent (during the last 3 months before the MPI study) acute coronary syndrome (ST elevation acute myocardial infarction, non-ST elevation acute myocardial infarction, or unstable angina). Additionally, patients with a medical history of an immunologic disease were excluded from the study unless they had stable disease (clinical remission) at the time of the CRP measurement.

### 2.2. Ethics

The protocol of this study was approved by the Hospital’s Clinical Research Committee. As this was a retrospective analysis of MPI SPECT data, no written patient consent was required. 

### 2.3. Study Protocol

The application of myocardial perfusion SPECT was performed according to the guidelines of the European Association of Nuclear Medicine (EANM) [26]. The MPI SPECT procedure was explained in detail to the patients. The same data acquisition parameters were used in all patients. A single-day protocol was used, with stress and rest images acquired in that order, approximately two hours apart [27,28]. Medical history information involved symptoms, classic risk factors for CAD like smoking behavior, hypertension, diabetes mellitus, dyslipidemia, obesity (considered if body mass index was greater than or equal to 30 kg/m^2^), and family history of CAD, and previous history of diagnosed cardiovascular disease and other health problems such as neurological diseases, rheumatological diseases, mental diseases, etc. 

Blood pressure and oxygen saturation were measured in all patients, and a 12-lead electrocardiogram was acquired. Maximal or symptom-limited treadmill exercise (Bruce protocol) was selected for patients capable of performing physical exercise; otherwise, an intravenous dipyridamole (0.56 mg/kg body weight) stress was applied, either alone or in combination with a single-stage 3 min Bruce treadmill exercise. All studies were performed using Technetium-99m tetrofosmin, an MPI radiopharmaceutical that was labeled in-house according to the manufacturer’s instructions.

### 2.4. Visual Analysis of Myocardial Perfusion SPECT

The recorded raw tomographic data under stress and rest were processed and reconstructed in three planes (short axis, horizontal long axis, and vertical long axis) according to established algorithms and assessed by two experienced board-certified nuclear medicine physicians, using a 17-segment polar map as previously reported [29,30] in stress and rest images. Each segment was scored on a scale of 0 to 4, depending on the degree of the perfusion deficit. Between the two readers, no serious inter-reader variability was seen. The differences were approximately ±2. A summed stress score (SSS) of over 3 was considered myocardial ischemia [SSS corresponds to either stress-induced or resting perfusion defects (i.e., ischemia + infarct)]. Myocardial ischemia was mild for SSS 4 to 8, moderate for SSS 9 to 13, and high if SSS was above thirteen [31]. Summed rest score (SRS) corresponds to perfusion defects at rest indicating probable myocardial scar. Furthermore, the summed difference score (SDS = SSS − SRS) is an important measurement since it denotes the presence of reversibility after exercise or pharmacologic stress.

### 2.5. CRP Measurements

All enrolled subjects had a serum CRP value available within one month from the MPI SPECT. For CRP measurements, the quantitative IMAGE^R^ 800 Immunochemistry System (Beckman Coulter, Brea, CA, USA) was used, which applies rate nephelometry and performs a patented dynamic blanking algorithm for selected analytes; the upper limit for CRP in our Institution is 6 mg/L.

### 2.6. Statistical Analysis

Continuous data are presented as mean ± standard deviation or median values (interquartile range), while dichotomous data are presented as numbers (percentage). Comparisons between the two groups of patients (high vs. low CRP) were made using the Fischer χ^2^ test for dichotomous variables, and Student’s *t*-test and the Mann–Whitney U test for continuous variables. General linear model analysis was used to detect potential interactions of CRP levels with other parameters and associations with SPECT findings. Linear and logistic regression analysis was implemented to identify independent predictors of SSS, SRS, and SDS treated either as continuous or dichotomous variables. A two-tailed *p*-value < 0.05 was used to determine significant associations. All analyses were performed using the software IBM SPSS Statistics version 21 (IBM, Armonk, NY, USA).

## 3. Results

Overall, 102 patients (69 male) from the medical records of the Nuclear Medicine Department of our Hospital met the inclusion criteria. Our population characteristics are presented in Table 1.

Among them, 69 patients were subjected to an MPI scan due to non-specific cardiac symptoms (workup to rule out CAD). The mean age of all participants was 71 years; 68% were males, and 32% had a history of “obstructive” macrovascular CAD (confirmed via a previous coronary angiography). The prevalence of classic risk factors was 77% for hypertension, 39% for diabetes mellitus, 64% for dyslipidemia, and 26% for smoking. Moreover, 26% of the population was obese, and 38% had increased CRP values (>6 mg/L). The median CRP value was 4 mg/L (2 mg/L, 10 mg/L) in the total population [2 (2, 3) mg/L in the low CRP group versus 12 (9, 23) mg/L in the high CRP group, *p* < 0.001)].

The SPECT study results revealed that 78% of the participants had a high summed stress score (SSS > 3), and 60% had a high summed difference score (SDS > 1). Patients with increased CRP had higher SSS (*p* = 0.006) and SRS (*p* = 0.001) compared to patients with low CRP. In addition, a higher risk for increased SSS > 3 was noted in patients with increased CRP (OR 9.25, 95% CI 2.03, 42.13, *p* = 0.001). However, no differences were observed in other studied clinical risk factors between the two groups (*p* > 0.05 for all comparisons) (Table 1). 

**Figure 1:**
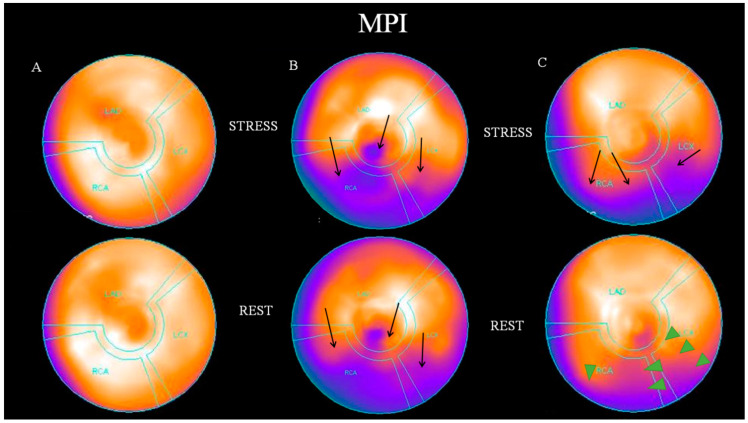
Three patients with different stress-rest bull’s eyes MPIs are depicted. (**A**) normal MPI and normal CRP; (**B**) abnormal MPI and high CRP; (**C**) abnormal MPI and high CRP; black arrows show defects; green arrowheads show reversible defects. MPI: myocardial perfusion imaging; SSS: summed stress score; SRS: summed rest score; SDS: summed difference score; CRP: C-reactive protein.

Figure 1 shows stress-rest bull’s eye images of three patients. Patient (A) had a normal MPI (SSS = 0) and a normal CRP value of 2 mg/L. Patients (B) and (C) had abnormal MPIs. The SSS of the second patient (B) was 19, SRS 17, and SDS 2, indicating severe myocardial ischemia with irreversible lesions. This patient had a high CRP of 24 mg/L. Finally, the SSS of the third patient (C) was 15, SRS 5, and SDS 10 (severe myocardial ischemia, with good reversibility—green arrows), and CRP was 11 mg/L.

**Figure 2:**
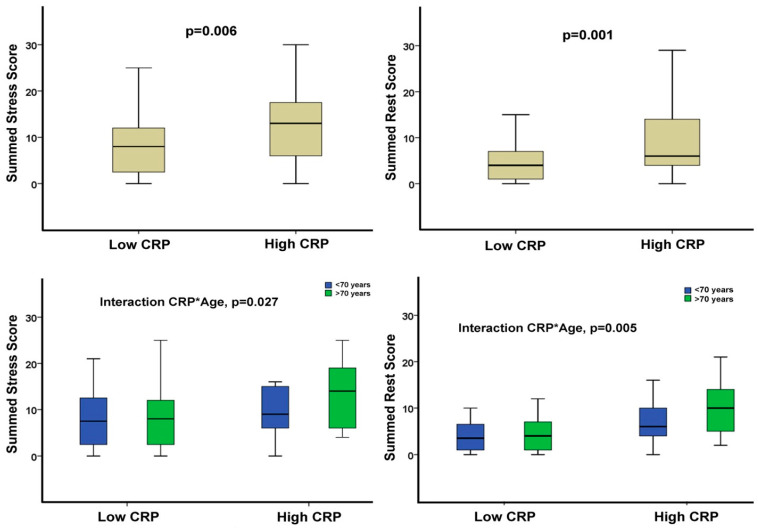
(**Upper left**): high CRP is associated with a higher SSS (*p* = 0.006) in overall population. (**Upper right**): high CRP is associated with a higher SRS (*p* = 0.001) in overall population. (**Lower left**): the correlation of increased CRP with a higher SSS (*p* = 0.027) is more evident in patients older than 70 years old. (**Lower right**): the correlation of increased CRP with a higher SRS (*p* = 0.005) is more evident in patients older than 70 years old. SSS = summed stress score SRS = summed rest score.

The association of SSS and SRS with CRP levels was more evident in older patients, i.e., >70 years old, *p* = 0.027 and *p* = 0.005, respectively, for interactions with age (Figure 2). No significant differences in SDS were shown between patients with increased versus low CRP levels.

In linear regression analysis, independent predictors of (1) increasing SRS were male gender (B 5.07, *p* < 0.001) and CRP > 6 mg/L (B 3.51, *p* = 0.005) (R^2^ 0.21, *p* < 0.001), and (2) increasing SSS, were male gender (B 5.48, *p* = 0.001) and CRP > 6 mg/L (B 3.32, *p* = 0.037) (R^2^ 0.15, *p* < 0.001). No studied variable was associated with SDS in linear regression analysis. In logistic regression analysis, independent predictors of (1) increased SSS (i.e., >3) were male gender (OR 6.53, *p* = 0.001) and CRP > 6 mg/L (OR 9.53, *p* = 0.005), and (2) was increased SDS (i.e., >1) and history of dyslipidemia (OR 2.47, *p* = 0.033). 

## 4. Discussion

Nuclear medicine is a medical specialty using radioactive isotopes for imaging and treatment, providing important disease information in several medical specialties, including cardiology [32,33,34]. One of the most widely used methods in nuclear cardiology is MPI SPECT, which may be used either for diagnosis or prognosis of various heart conditions [20,23]. 

MPI SPECT represents a precision medicine tool able to accurately differentiate between normal and abnormal processes inside the heart [35,36]. Various radiotracers may be used for the MPI SPECT, such as technetium-99m tetrofosmin or technetium-99 m sestamibi, which are dispersed passively within the myocardium depending on their viability, and thallium-201, which is distributed actively within the myocardial cells. These radiotracers were injected, and pictures were taken both at rest and during stress provoked either by exercise or pharmacologically. In cases where the radiotracer uptake was normal both at rest and exercise, the examination was normal, denoting healthy myocardial tissue. When there was poor uptake post-exercise and improved uptake during rest, the examination indicated a reversible perfusion defect. If the uptake was poor during exercise and did not improve at rest, the examination indicated that the myocardial tissue was constantly injured, such as after a myocardial infarction [20]. MPI, apart from detecting coronary artery disease, can assess the myocardial status of patients with coronary microvascular dysfunction, which represents an abnormal vasoconstrictor/vasodilatory coronary capability [37]. 

CRP represents a greatly conserved plasma protein involved in the inflammatory process [12], consisting of five subunits that bind to phosphocholine in a Ca^2+^-dependent manner. It represents an acute-phase plasma protein and a constituent of the acute-phase response [38]. Recent evidence indicates that atherosclerosis is linked to vascular inflammation involving both innate and adaptive immune responses and the mobilization of monocytes, macrophages, neutrophils, and lymphocytes [39]. A prospective study of 316 patients with STEMI managed with primary PCI reported that the estimated CRP velocity, defined as the value of the first CRP measurement divided by the time from the patient’s first reported symptom, was associated with microvascular infarct pathology [40]. A strong relation between CRP and the future risk of a fatal or non-fatal coronary event was found. A 1-SD increase in the log-transformed value of CRP was associated with a remarkable 50% increase in coronary risk, and subjects in the highest quintile of the CRP distribution thus showed a 2.6-fold increase in their risk of a future coronary event [41]. Furthermore, local and systemic inflammation is gaining ground as a potential contributing mechanism in vasospastic angina [42], especially the inflammation associated with the perivascular adipose tissue [43]; CRP might be a useful biomarker in that direction. 

However, apart from these, in many studies, raised CRP values due to subclinical infections cannot be excluded. An infectious pathogenesis theory as a causative background of atherosclerosis has also been suggested [44].

In the current study, we studied the association between increased CRP values and abnormal MPI findings. A significant association of impaired myocardial perfusion in SPECT imaging with prevalent inflammation (as assessed by increased CRP levels, i.e., >6 mg/L) was shown; this effect was more prominent in older patients (>70 years old). Subclinical inflammation is an integral part of the atherosclerotic process and its progression and has been previously associated with increased risk for cardiovascular events [45,46]. Whether levels of inflammation may be related to the presence of myocardial ischemia in patients with symptoms suggestive of CAD is a matter of ongoing research. A limited number of studies have reported a significant positive association of inflammation with MPI SPECT ischemia findings, although not consistently [22,24,25]. 

In patients with symptomatic angina or a history of myocardial infarction, the extent of stress-induced myocardial ischemia in SPECT was higher in case of increased CRP levels (approximately 5-fold increased risk); high CRP levels predicted both exercise-induced ischemia and outcome [22], while in patients with stable CAD who underwent SPECT imaging and/or new regional wall motion abnormality on transthoracic echocardiography during mental stress, each 1 mg/L increase in CRP was associated with a 20% higher risk of reversible perfusion defect/ischemia (in univariate logistic regression analysis) [47] and this relationship remained in multivariate analysis. The increasing risk for positive ischemic tests across tertiles of CRP demonstrated a non-significant trend. However, we must take into account that non-cardiac stress-related disorders may be associated with higher inflammation markers (including CRP) and greater risk of cardiovascular diseases [48,49]. In high-risk asymptomatic patients (based on the European SCORE model) without known CAD, hs-CRP has been found to be an independent predictor of myocardial perfusion defects and has been suggested to be used as a criterion to guide myocardial SPECT imaging [30], which also seems to apply for patients with chronic kidney disease [50,51,52]. Majstorov et al. showed that in patients with confirmed or suspected CAD, those patients with mild or moderate perfusion defects in SPECT had significantly higher CRP values (2.7 mg/L vs. 4.2 mg/L, *p* = 0.01) compared to patients with normal or near normal myocardial perfusion [53]. In contrast to these results, Rathcke et al. showed that in a population of patients with an intermediate risk of having CAD or with a known history of CAD, those subjects with abnormal MPI had similar CRP levels with those with normal MPI [54]. Interleukin-6, YKL-40, and N-terminal fragment of the prohormone brain natriuretic peptide levels were significantly elevated in the group with abnormal MPI [53,54]. Recently, another index, i.e., the ratio of two inflammatory markers (CRP to albumin; CAR), was investigated for the prediction of ischemia. In multivariate analysis, CAR showed the best capability to discriminate myocardial ischemia detected by MPI [55]. Finally, whether changes in inflammatory indices may result in changes in SPECT imaging is not known. However, results from the PLATO Trial have shown that one month after myocardial infarction, the degree of change (decrease) of hs-CRP is more pronounced in patients without obstructive compared to patients with obstructive CAD [56]. 

We found that the association of SSS and SRS with CRP levels was more evident in older (>70 years) patients. This may be explained by the higher risk of CAD in the elderly population since age is the strongest risk factor related to CAD [57]. We could hypothesize that the possibility of this association to be significant is greater when the areas of ischemia in MPI are longer and more frequent. Regarding the validity of the method in the elderly, Rai et al. evaluated the optimal non-invasive stress tests in elderly patients [58]. In their meta-analysis, the test with the greater risk (abnormal compared to normal stress) of future cardiac events was found to be MPI. These results indicated that MPI accurately stratified risk in the elderly [59]. Moreover, a second hypothesis could be that our result is the effect of confounding bias. Baseline CRP or hs-CRP levels increase with aging [59,60,61,62], and therefore, both higher levels of CRP and the presence of ischemia in MPI could be the consequences of aging. In that case, it would be expected that MPI cannot have any prognostic value that is not truthful [58]. 

The predictive role of CRP has also been investigated in relation to other myocardial perfusion imaging modalities. In patients with microvascular dysfunction and ischemia and no obstructive coronary arteries (INOCA) who underwent positron emission tomography, those who had CRP > 3 mg/L had a more severe impairment of coronary flow reserve (2.14 ± 0.33 vs. 3.16 ± 0.76; *p* = 0.001); furthermore, a negative correlation between CRP levels and coronary flow reserve (*R* = −0.49, *p* = 0.02) was shown [63]. In patients with typical angina pectoris without CAD, increased CRP levels were associated with an impaired myocardial perfusion reserve index following stimulation with intravenous adenosine and intracoronary acetylcholine in cardiac magnetic resonance [64]. 

According to the last published survey, the total number of myocardial perfusion scintigraphy studies in European countries was lower than invasive coronary angiographies (ICAs) during the same time period [65,66]. Despite patients with positive results on MPI being significantly more likely to have obstructive CAD [as assessed by invasive coronary angiography (ICA)] than those who did not undergo non-invasive testing, MPI is performed in only one out of four patients before elective ICA [67]. If we take into consideration that most (but not all) asymptomatic or stable CAD patients have a benign prognosis and they would be treated conservatively [67], it is essential not only to increase MPI diagnostic yield but also to find additional markers in combination with MPI results that optimize the impact on prognosis of MPI. Can CRP or other inflammatory indices be used in combination with MPI for these purposes? Does combined testing (MPI and CRP) improve the probability of obstructive CAD? Would the addition of an inflammatory marker beyond MPI results help us to make a decision about performing or not angioplasty and stenting in an angiographically non-significant (or even significant) lesion? The hypothesis is that in patients with ischemia in MPI, inflammatory indices are higher if coronary artery plaque burden is higher in angiography or (even better) intravascular imaging. There are no data about this hypothesis, and studies to address this area are needed. Nowadays, the invasive evaluation of ischemia by means of physiological assessment remains the gold standard for percutaneous coronary intervention decision-making [68], although it is still (as the MPI testing) underused in real-life clinical practice [69,70]. 

### Limitations

Our study was retrospective and included a limited number of variables in association with SPECT findings. CRP values were not acquired on the same day of the SPECT in most cases but rather assessed on a close date to the exam. Furthermore, we did not use an hs-CRP assay, which is the case in most of the previous epidemiological studies; hence, results may be different in studies using different assays. Another important limitation is the fact that we did not have an invasive coronary angiography to associate CRP values with the existence and extension of CAD. Finally, we used the cut-off value of 6 mg/L to distinguish patients with high or low CRP values. Although other values have been used (and patients with CRP levels between 3 and 6 mg/L are not considered as they have normal values from many investigators), other studies showed that this value may have the best prognostic value [71]. 

## 5. Conclusions

We investigated the association of serum CRP levels with MPI SPECT findings in a population of patients with suspected or stable CAD. A high level of CRP has been found to be associated with the presence and extent of stress-induced myocardial ischemia in MPI SPECT, indicating that subclinical inflammation might be associated not only with the presence of coronary atherosclerosis but also with the severity of coronary plaque burden and clinical manifestation of ischemia. Larger prospective studies are needed to examine whether indices of subclinical inflammation add diagnostic value to an MPI SPECT study so that they would be part of a diagnostic workup for CAD.

## Figures and Tables

**Table 1:** Population characteristics and association of CRP levels with various variables.

	Total Population N = 102	CRP ≤ 6 mg/L N = 63	CRP > 6 mg/L N = 39	*p* Value
Age, yrs	71 ± 11	69 ± 11	72 ± 11	0.189
Male gender, n (%)	69 (68)	40 (64)	29 (74)	0.254
CAD Hx, n (%)	33 (32)	21 (33)	12 (31)	0.788
Hypertension, n (%)	78 (77)	50 (79)	28 (72)	0.381
DM, (%)	40 (39)	24 (38)	16 (41)	0.768
Dyslipidemia, n (%)	65 (64)	42 (67)	23 (59)	0.432
Obesity, (%)	26 (26)	16 (25)	10 (26)	0.978
Smoking (%)	26 (26)	16 (25)	10 (26)	0.978
SSS	8 (4, 15)	8 (2, 12)	13 (6, 18)	**0.006**
SRS	5 (2, 9)	4 (1, 7)	6 (4, 14)	**0.001**
SDS	2 (1, 6)	2 (0, 5)	3 (1, 6)	0.703
SSS > 3	79 (78)	42 (67)	37 (95)	**0.001**
SDS > 1	61 (60)	37 (59)	24 (62)	0.779
CRP, mg/L	4 (2, 10)	2 (2, 3)	12 (9, 23)	**<0.001**

CAD: coronary artery disease, DM: diabetes mellitus, SDS: summed difference score, SRS: summed rest score, SSS: summed stress score. The bold indicates the statistical significance.

## Data Availability

The data presented in this study are available on request from the corresponding author. The data are not publicly available due to privacy issues.
